# Healthcare utilization and outcomes of patients discharged to skilled nursing facilities on parenteral antimicrobial therapy

**DOI:** 10.1017/ash.2025.171

**Published:** 2025-06-11

**Authors:** Madeline Langenstroer, Christopher Crnich, Sally A. Jolles, Jon P. Furuno, Brie N. Noble, Rosemary C. Bailey

**Affiliations:** 1 Department of Medicine, University of Wisconsin School of Medicine and Public Health, Madison, WI, USA; 2 William S. Middleton VA Hospital, Madison, WI, USA; 3 Department of Pharmacy Practice, Oregon State University College of Pharmacy, Portland, OR, USA; 4 Department of Biostatistics, Mayo Clinic, Scottsdale, AZ, USA

## Abstract

Hospitalized patients are commonly discharged to skilled nursing facilities (SNFs) on outpatient parenteral antimicrobial therapy (OPAT). In this study, 101 patients discharged to a SNF on OPAT required considerable post-hospital care coordination and experienced high readmission and mortality rates within 90 days, contributing to literature characterizing OPAT patients in other settings.

## Introduction

Outpatient parenteral antimicrobial therapy (OPAT) is a common aspect of treating complicated infections.^1^ While a substantial volume of post-hospital OPAT is delivered in skilled nursing facilities (SNF-OPAT), outcomes studies have largely focused on patients receiving OPAT in an infusion clinic or at home.^2–4^ We conducted a retrospective cohort study focused on hospitalized patients discharged on SNF-OPAT. We hypothesized that SNF-OPAT patients would experience high rates of complications and healthcare utilization.

## Methods

Using electronically extracted data from a larger multisite cohort study,^5^ we conducted a nested cohort study of patients hospitalized at an academic Midwest tertiary care referral center who were discharged to a SNF on oral and/or parenteral antimicrobials from 1/1/2016 to 12/31/2018. Patients discharged with OPAT intended to last >2 weeks are routinely followed by the facility’s Infectious Disease (ID) service. Patients discharged on SNF-OPAT who were ≥18 years of age, had been seen by an ID specialist during their hospitalization, and were scheduled for follow-up in the ID Clinic were included. Patients who completed antimicrobial therapy while hospitalized did not require ID Clinic follow-up, or had follow-up at another facility were excluded.

We performed an electronic health record review of eligible patients to collect data on demographics, length of index hospitalization, primary ID diagnoses, inpatient and post-discharge antimicrobial therapy, and ID clinic care coordination and face-to-face encounters. We reviewed emergency department (ED) visits (not resulting in hospitalization), hospital readmissions (including the direct and ED-initiated), and mortality in the 90 days following the index hospitalization. We characterized OPAT modifications by class, route, and reason. Post-hospital healthcare utilization was characterized by the number of phone calls between staff in the ID clinic and SNFs, and face-to-face encounters in ID and non-ID sub-specialty clinics. ED, admission, and mortality notes were reviewed to determine the relationship between these outcomes and the primary ID diagnosis (infection-related) or OPAT (treatment-related).

We performed descriptive analysis using Microsoft Excel. The University of Wisconsin School of Medicine and Public Health Institutional Review Board approved this study with a waiver for informed consent.

## Results

A total of 155 SNF-OPAT patients hospitalized during the study period were scheduled to be discharged. Fifty-four patients were excluded for: (1) lack of follow-up need (n = 20); (2) receipt of post-hospital care at another facility (n = 17); (3) completion of therapy before discharge (n = 10); and (4) lost to follow-up (n = 7). The final study sample consisted of 101 patients. Two-thirds of patients were over the age of 65; 58% were male; and most were non-Hispanic white (Table 1). The most common primary ID diagnoses included 39 bone/joint infections and 24 endovascular infections (Table 1).

**Table 1.** Patient demographics, treatment characteristics, and healthcare utilization

PATIENT CHARACTERISTICS		
	Number	%
***Patient Demographics***		
Age ≥ 65	68	67.3
Male	59	58.4
White	97	96.0
***Infection Types***		
Bone/Joint	39	38.6
Endovascular	24	23.8
Skin and Soft Tissue	17	16.8
Intra-abdominal	12	11.9
Other ^a^	9	8.9
***Treatment Modifications***		
Total	68	ref
Step down, chronic suppression, or prophylaxis	35	51.5
Adverse medication reactions	15	22.1
Addition of second antimicrobial	9	13.2
Additional culture results available	5	7.4
SNF unable to continue providing preferred agent	4	5.9
**HEALTHCARE UTILIZATION**		
	Number	Mean (SD)
***Index hospitalization length of stay (LOS)***	---	11.4 (6.2)
***Post-discharge healthcare utilization***		
Infectious disease (ID) clinic follow-up encounters	169	1.67 (1.0)
Non-ID sub-specialty clinic follow-up encounters	225	2.7 (2.6)
ID clinic telephone encounters	1370	13.4 (7.2)

^a^Other diagnoses include fever, altered mental status, sinusitis, pneumonia, and empyema.

^b^Value reported as a mean in days with (standard deviation).

^c^Values reported as a total and mean with (standard deviation).

Patients received 4,716 days of SNF-OPAT, with a median duration of 48 treatment days (IQR: 25.5 d). The most common antimicrobial classes prescribed included penicillins (33%) and cephalosporins (31%). The initial antimicrobial regimen was modified at least once in 44 patients (46.5%) and two or more times in 19 patients (19%) for a total of 68 treatment modifications. Approximately half (n = 35) of the modifications were for IV to oral step-down, suppressive, or prophylactic therapy. The second most common treatment modification (Table 1) was an adverse reaction (n = 15) to the index antimicrobial regimen with renal dysfunction and generalized rash most cited.

Healthcare utilization within 90 days of index hospitalization was substantial. There were 424 face-to-face sub-specialty encounters with 89.9 visits per 1000 days of follow-up (DFU). Patients had on average 4.2 encounters (SD: 2.86). Follow-up in ID clinic (36.8 per 1000 DFU) accounted for 40% of these encounters (mean: 1.7/subject, SD: 0.99). The median time from hospital discharge to initial ID clinic follow-up was 22 days (IQR: 15). ID clinic nursing staff conducted 1,370 telephone encounters (290 per 1000 DFU) leading to an average of 13.4 calls per patient (SD: 7.2) (Table 1).

Seventeen patients had 16 ED encounters within 30 days of hospital discharge, and 25 ED encounters within 90 days. Five patients had two or more ED encounters. Nearly half of these encounters were for OPAT-related reasons (Table 2). The readmission rate at 30 days was 29%. Fifty-two hospital readmissions occurred among 39 patients within 90 days of the index hospitalization. One patient had 3 readmissions. Approximately 17% of readmissions were OPAT-related, and another 33% were infection-related (Table 2). Five patients died within 30 days of hospital discharge and the 90-day mortality rate was 8.9% (Table 2).

**Table 2.** SNF-OPAT Outcomes within 30- and 90-days post-discharge

	30-days post-discharge			90-days post-discharge		
	*N*	%	Per 1000 SNF-OPAT Days	*N*	%	Per 1000 SNF-OPAT Days
***Emergency Department Visits***	16		5.3	25		5.3
Treatment-related	8	50.00	2.6	12	48.0	2.5
Infection-related	3	18.8	1.0	4	16.0	0.8
Non-treatment, non-infection-related	5	31.3	1.7	9	36.0	1.9
***Hospital Readmissions***	29		9.6	52		11
Treatment-related	9	31.0	2.8	9	17.3	1.9
Infection-related	8	27.6	2.6	17	32.7	3.6
Non-treatment, non-infection-related	12	41.4	4.0	26	50.0	5.5
***Deaths***	5	5.0	1.7	9	8.9	1.9

## Discussion

The current study represents one of few studies focusing explicitly on outcomes among SNF-OPAT patients and shows that healthcare system utilization and adverse health outcomes of these patients are high. The largest US-based OPAT studies published since

2000^1^,^6–8^ primarily focused on outcomes among patients receiving OPAT at home or an infusion center. The 30-day (29%) readmission rate identified in the current study was substantially higher than the 30-day readmission rates (<20%) reported in three studies (n = 651) recovered from our review.^6–8^ The 30-day mortality rate observed in the current study (5%) is similar to that reported in studies recovered from our review (4.5%–6.1%).^7^ While a previous study found no statistically significant difference in mortality rate at 30 and 90 days, the 90-day mortality rate observed in the current study was twice as high (8.9%).^7^

The high rate of adverse outcomes observed among SNF-OPAT patients in the current study may not be surprising, given these patients are older and have more comorbidities compared to patients receiving OPAT in other settings. Nearly half of the ED encounters and readmissions observed were for OPAT- or infection-related factors, which are partially avoidable with close follow-up.^9^ A high volume of contact between the ID clinic and SNF nursing staff was identified in this study. However, the average face-to-face follow-up interval of 3 weeks (median: 22 days, IQR: 15) suggests a low intensity of direct contact between patients and providers. A recent study demonstrated a lower risk of hospital readmission for patients whose OPAT follow-up was conducted by an ID-trained provider rather than a nurse but highlighted a statistically significant decrease in those discharged to SNF.^9^ Although the SNF setting should theoretically provide a “safer” environment for OPAT, our findings question whether shorter interval follow-up can positively influence clinical outcomes such as readmission rates. While there are clear logistical and financial barriers to more frequent follow-up with this patient population, more liberal use of telehealth is an approach that may enhance the safety of SNF-OPAT, though existing regulations and payment structures remain uncertain.^10^

The current study has several limitations, including a relatively small study population recruited from a single medical center and an inability to evaluate outcomes among patients who received post-discharge follow-up at another facility or were lost to follow-up. Additionally, study data was from 2016–2018. The intensity of post-hospital ID follow-up may have increased with wider implementation of telemedicine in SNFs during the COVID-19 pandemic and through expansion of other communication modes. Lastly, the absence of a comparator for non-SNF-OPAT ED visits, readmissions, face-to-face follow-up, and telephone encounters (including reason for contact and time spent) and lack of comprehensive outpatient care coordination evaluation present additional limitations.

The current study suggests that an intervention worth studying in SNF-OPAT patients includes closer post-discharge ID follow-up although future studies that directly compare outcomes of age- and comorbidity-matched patients receiving OPAT in other settings are needed.
